# PLACEMAKING AS CIVIC PARTICIPATION: A STORY OF AGENCY AND RESISTANCE AMONG BLACK AND LATINX IMMIGRANT OLDER ADULTS

**DOI:** 10.1093/geroni/igac059.1650

**Published:** 2022-12-20

**Authors:** Laurent Reyes

**Affiliations:** UC Berkeley, Berkeley, California, United States

## Abstract

Placemaking is a collective process that happens every day. However, little has been written about the ways older African Americans and Latinx immigrants create place in their everyday lives. Less has been said about the ways these efforts are examples of civic participation in the context of systemic inequality and oppression. Results from this phenomenological study that applies an intersectional life course perspective shows how older Black and Latinx immigrant adults maintain, preserve, and build their place in a society that continuously threatens their erasure. These results shine light on the ways Black and Latinx communities work to maintain the physical space (i.e., community safety and sanitation), preserve their culture and history, and build structures to ensure access to resources that will empower their communities. Their efforts across the micro, meso, and macro social environments and across time, demonstrates a concerted effort towards creating a dignified and just place in our society to nourish and sustain future generations.

